# Scientific Perspectives on Evaluating Fetal Physical Development Around Birth: Historical Approaches and Practices in Japan

**DOI:** 10.1111/jog.70324

**Published:** 2026-05-12

**Authors:** Norio Shinozuka

**Affiliations:** ^1^ Laboratory for Fetal Medicine Research Hiratsuka Kanagawa Japan

**Keywords:** estimated fetal weight, fetal biometry, growth chart, reference standard

## Abstract

Fetal growth assessment has been performed using ultrasound measurements and the estimated fetal weight (EFW) derived from them. The Shinozuka formula—a rational equation based on fetal volume and specific gravity—has served as the de facto standard, maintaining high accuracy across a wide range of gestational ages and independent of fetal body proportions. The reference values used to evaluate these measurements were established from a normal control survey population, representing fetuses developing under optimal conditions. As the distribution of these reference values has been confirmed to follow a normal distribution, measurements can be uniformly evaluated in relation to gestational age using standard deviations from the mean. This paper provides an overview of methods for fetal growth assessment, the theoretical basis of reference values, and the historical development and clinical application of fetal biometric evaluation in Japan.

## 1 Introduction; Historical Background of Fetal Physiological Growth Assessment in Japan

Birth size, weight, and height have historically been used as indicators for assessing a newborn's health at birth and predicting their prognosis. Since these references used actual birth population data, including data on preterm infants, they did not accurately represent intrauterine growth; however, prior to the development of accurate methods for estimating fetal weight (EFW), growth curves on such birth data were often referred to as intrauterine fetal growth curves. In Japan, there are reports by Funakawa [1], Nishida [2], Shinozuka [3], Ogawa [4], and Itabashi [5].

Western reports include those by Lubchenco [6], Brenner [7], Alexander [8], and Intergrowth 21 (Oxford) [9].

Ultrasound diagnostics were introduced into general obstetric practice from the late 1970s to the early 1980s. In the field of obstetrics, ultrasound was performed primarily for the following purposes.

1. Confirmation of pregnancy: Verification of the gestational sac (GS) and/or embryo.
2. Assessment of fetal growth and the intrauterine environment.: Confirmation and correction of the estimated date of delivery and gestational age at early pregnancy. Assessment of fetal growth using ultrasound measurements and EFW.
3. Screening for maternal and fetal abnormalities associated with pregnancy.

Since the publication of Nishida's growth curve [2] in 1984, various methods for estimating fetal weight using ultrasound have been developed. The concept involves estimating weight and using these birth weight charts to evaluate intrauterine growth. In the West, Walsof [10] and Hadrock [11] formulas were frequently used; however, both were regression equations based on actual data. Therefore, they had limitations in terms of providing consistent estimates with minimal error across a wide population—that is, accounting for variations in gestational age and fetal body proportions. To address these various issues, the Shinozuka formula [12] (1987) was developed; details will be discussed later.

By the 1990s, it had become common practice in Japan to conduct more than a dozen prenatal checkups and multiple ultrasound examinations during pregnancy, creating a need for reference values to evaluate ultrasound measurements and EFW over time.

While the definition of the population for growth assessment and the clinical evaluation methods will be explained in the next section, standard values for measurements and EFW were published in 1996. Those represented normal control values based on the assumption of normal growth under ideal in utero conditions. This report was approved by academic societies of the Japan Society of Ultrasound in Medicine (JSUM) in 2003 and by the Japan Society of Obstetrics and Gynecology (JSOG) in 2005 as standard values for Japanese fetal ultrasound measurements.

In 2012, the EFW reference values were included in the Official Maternal and Child Health Handbook as “fetal growth curves.”

In 2010, Revised “Neonatal Anthropometric Charts for Gestational Age at Birth” by Itabashi was published.

## 2 The Chronological Process of Human Physical Development

**FIGURE 1:**
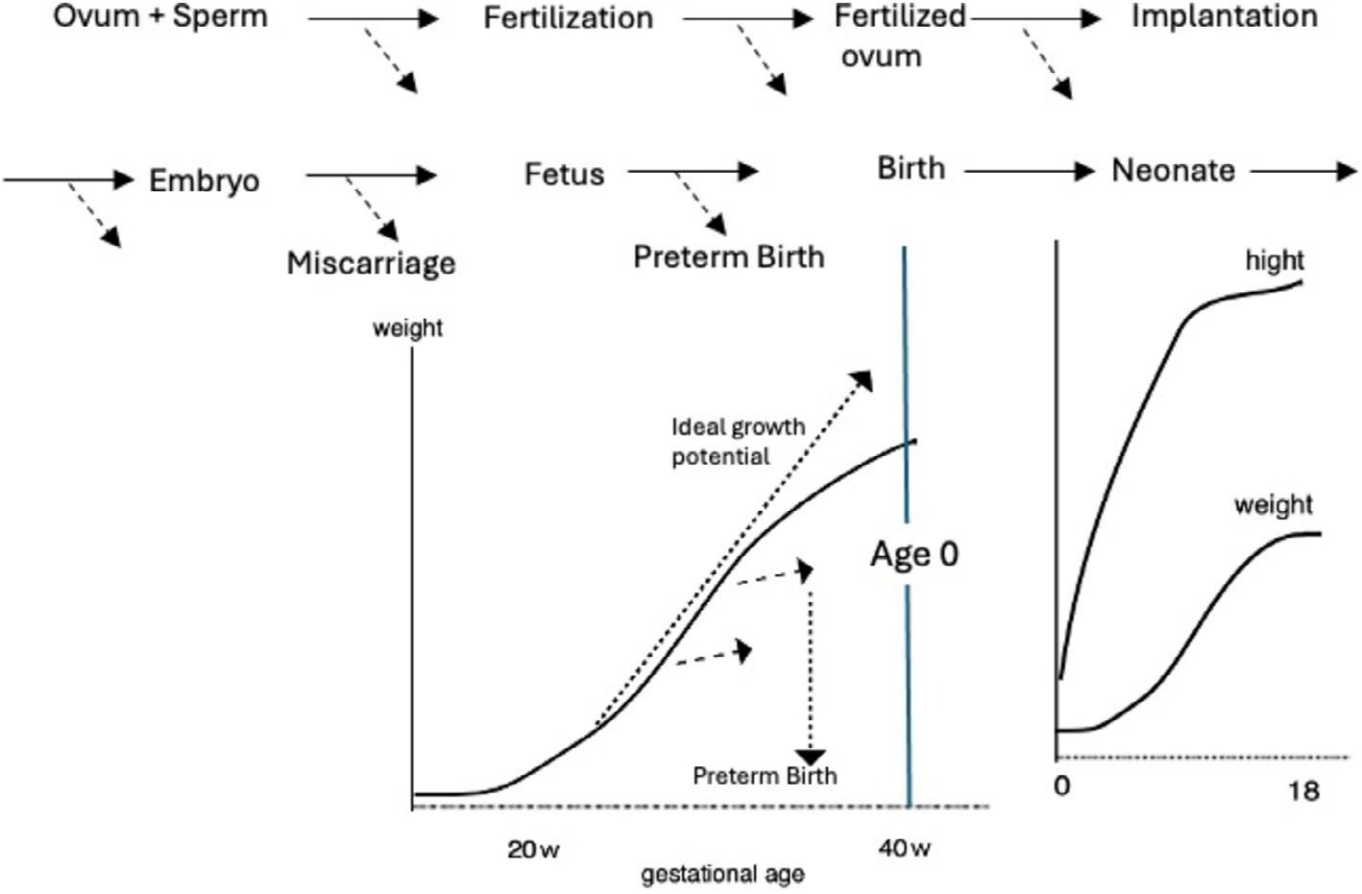
Schematic diagram of developmental process in early life. Dropouts (dashed arrow line) from the normal course during the developmental process. As a result of survival challenges due to various factors, the overall growth pattern takes an S‐shaped form.

The process of human growth leading up to birth can be characterized as a series of survival challenges, beginning with the fertilization of the sperm and ovum, followed by implantation, the embryonic stage, and the fetal stage, concluding with birth (Figure 1).

At birth, the time axis resets to age 0.

As a result of various influencing factors, the overall growth curve takes an S‐shaped form; however, some argue that the ideal growth potential up to the second trimester can be assumed to be linear.

## 3 Physical Growth Assessment in Fetus and Newborn

**FIGURE 2:**
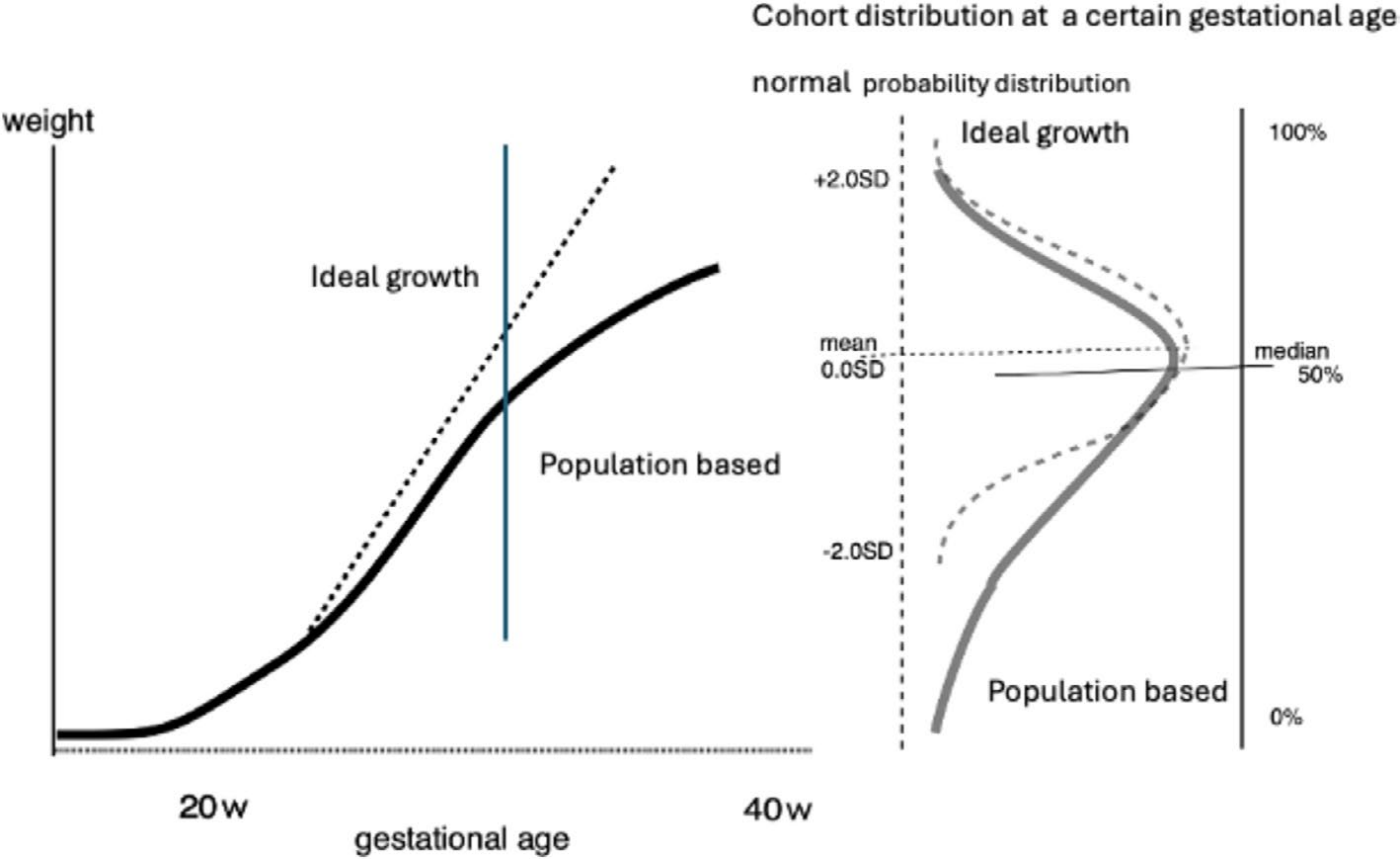
Concept of a reference cohort to configure for growth assessment in fetus and newborn. Population‐based cohort shows skewed distribution toward the lower end. Normal control cohort in ideal growth environment can be assumed to be normal probability distribution.

When we evaluate measurement data, what population should we use as a reference? The distribution of subjects at a certain gestational age is shown in Figure 2. Considering the growth process described above, the overall shape of the distribution skewed toward the lower end. This cohort, namely population‐based cohort, includes all data, both normal and abnormal cases, and the evaluation is conducted using percentiles form.

On the other hand, the cohort in an ideal environment—that is, the normal control group—is generally assumed to follow a normal probability distribution. Thus, the subject can be evaluated based on its deviation from the mean.

Definition of the survey population cohort used as the reference for evaluating measured values is shown in the Table 1.

**TABLE 1:** Definition of the reference survey population cohort for evaluating measured values.

(a) Population‐based group
A population comprising all group data, including abnormalities
Evaluates relative position within the population
Does not follow a normal distribution
(b) Normal control group
A group within the population that can be judged as normal (ideal) data
Appropriate conditions for defining “normal” are required
The normal probability distribution can be assumed based on statistical indicator such as skewness and kurtosis
Determining whether the data under evaluation is normal or not
(c) Population of descriptive statistics (reference values)
A population selected and extracted from population‐based data that exhibits minimal variation; it includes a certain outlier that occurs universally
Does not follow a normal distribution

In the evaluation of newborns, actual measurements at birth are used, so the cohort is population‐based as shown in Table 2a.

**TABLE 2:** Criteria for defining a normal developed fetus in an ideal intrauterine environment.

(1) Cases where gestational age and due date validity are assured (singleton pregnancies) confirmed by early ultrasound (crown rump length (CRL)/bi‐parietal diameter (BPD))
(2) Cases without pregnancy complications or maternal complications
(3) Cases without fetal malformations or abnormalities
(4) Cases with term delivery, normal birth weight (appropriate for gestational age [AGA]), normal outcomes, and an uncomplicated neonatal prognosis.

Fetal physical growth is evaluated based on ultrasound measurements and the EFW calculated from these measurements. From a clinical perspective, normal controls are preferred as the comparison group for prospective growth assessment. A strict definition is required for the selection of normal controls, and the Shinozuka/JSUM2003 criteria were developed based on this principle. However, many Western fetal growth references employ the descriptive survey population cohort [13, 14] described in Table 2c, possibly due to the difficulty in defining and collecting normal reference values [15].

## 4 Standard Reference Values for Ultrasound Fetal Growth Assessment in Japan

In the early days of clinical ultrasound, growth charts like those in Table 2c, which compile general data for reference, were commonly used. The Shinozuka/JSUM2003 criteria were established as normal reference values under ideal conditions, with the aim of enabling early detection of fetal growth abnormalities and confirming normal growth [16, 17].

The conditions shown in Table 2 were set as a method for accumulating ultrasound measurement data that can be assumed to represent “normal survey control cohort” in utero. Although the details have been reported elsewhere, the current standards for fetal measurements in Japan were established by compiling cases that satisfy these criteria, analyzing their distribution by gestational age, statistically confirming normality, and then constructing nomograms for the mean and standard deviation. Because the normality of the reference data is ensured, ultrasound measurements and EFW can be standardized using the standard deviation appropriate for each gestational age. Basic fetal measurement standards other than BPD, abdominal circumference (AC), and femur length (FL) (e.g., long bone measurements) have been established using the same methodology. However, for evaluation methods based on ratios of measured values—such as blood flow parameters, which typically do not follow a normal probability distribution, results are expressed as percentiles.

## 5 Equation for Fetal Weight Estimation

In the past, a vast number of fetal weight estimation methods have been devised. While Hadlock's formula is commonly used in Europe and the United States, this section provides an overview of the method recommended by Japanese academic societies (Shinozuka/JSUM) and its theoretical background. Fundamentally, this method employs multiple regression analysis, with fetal weight as the dependent variable and multiple ultrasound measurements, such as BPD, as independent variables. With such equations, depending on the bias of the cases used to create them, they possess adequate estimation accuracy for normal cases and fetuses at advanced gestational ages, but accuracy is low in cases with abnormal development or fetuses with different proportions. Furthermore, because it is fundamentally an application of the least squares method, variables with low measurement error, such as BPD, have a high contribution rate, leading to a skewed distribution of overall accuracy; thus, there are limitations inherent in the regression analysis methodology.

**FIGURE 3:**
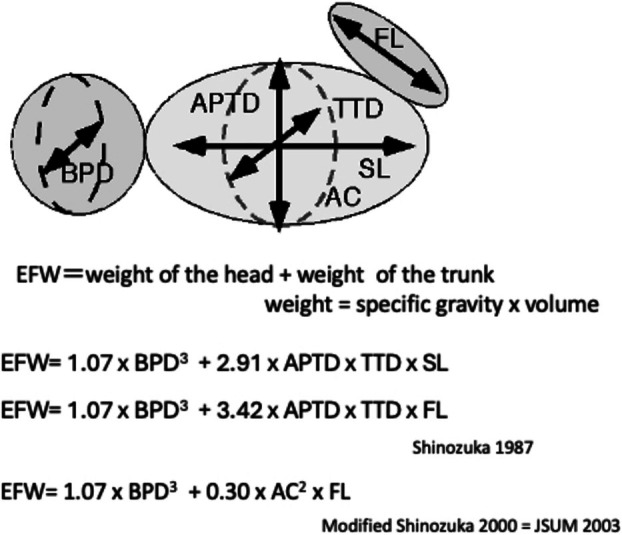
Fetal weight estimation formula based on a theoretical fetal volume model. Actual measurement values of neonatal volume and specific gravity were applied for theoretical formulas. BPD (biparietal diameter), APTD (antero‐posterior trunk diameter), TTD (transverse trunk diameter), AC (abdominal circumference), SL (spine length), FL (femur length). Modified citation form Shinozuka et al. [16].

Shinozuka/JSUM formula was developed to improve estimation accuracy for fetuses with different proportions, such as those with fetal growth restriction (FGR) or small fetuses, and to estimate weight within a consistent margin of error that is not biased toward specific gestational ages or weights. In other words, it is a theoretically meaningful formula built to accurately estimate population‐wide outcomes [12] (Figure 3).

EFW equations are as follows [2].

$$\mathrm{EFW}=1.07\times {\mathrm{BPD}}^{3}+3.42\times \text{APTD}\times \mathrm{TTD}\times \mathrm{FL}$$

$$\mathrm{EFW}=1.07\times {\mathrm{BPD}}^{3}+0.30\times {\mathrm{AC}}^{2}\times \mathrm{FL}$$

The formula is a theoretically derived equation rather than an ordinal regression model based on ultrasound measurements. Instead, it is constructed using a fetal model grounded in empirical data on neonatal specific gravities and volumes. In this equation, the first term represents the weight of the head, while the second term represents the weight of the trunk. The original parameter, defined as the product of APTD and TTD, was substituted with AC based on an analysis of the relationships among the measurement parameters. As this method is grounded in theoretical equations related to fetal volume, the measured parameters are mathematically interchangeable [17].

Prospective studies have demonstrated that these formulas achieve high accuracy in estimating fetal weight across a wide range of cases, regardless of whether FGR presents symmetrically or asymmetrically [12] (Table 3). Prior to its adoption as the standard formula in Japan, a comparative study conducted by JSUM demonstrated the superiority of the Shinozuka formula over other methods (Table 4).

**TABLE 3:** Range of error related to actual birth weight (prospective study).

Birth weight (gm)	≤ 5%	≤ 10%	≤ 20%
0–999	33.3%	61.1%	88.9%
1000–1499	33.3%	72.7%	97.0%
1500–1999	45.5%	70.7%	96.2%
2000–2499	40.3%	72.7%	96.4%
2500–2999	47.1%	71.6%	94.8%
3000–3499	35.7%	68.2%	94.3%
3500—	45.3%	81.8%	97.0%
Light for date only	42.7%	79.8%	98.1%
			*N* = 657

**TABLE 4:** Comparison of the accuracy of different formulas.

*N* = 479	Error for estimated weight (%)		Error for birth weight	
	Mean	SD	Mean	SD
Warsof	−0.07	12.36	0.65	11.78
Deter	4.33	11.26	5.88	11.81
Eik‐Nes	−1.36	14.73	0.39	14.01
Shepard	4.08	11.52	5.68	12.17
Jordaan	6.28	10.66	8.01	12.27
Aoki	2.43	9.12	3.38	9.56
Hadlock	−0.03	10.74	1.01	10.65
Miyamoto	9.39	10.42	11.63	11.54
Woo	−11.04	11.82	−8.99	9.08
Shinozuka	−0.14	9.71	0.79	9.67

*Note:* JSUM committee.

EFW reference values were included in the Official Maternal and Child Health Handbook as “fetal growth curves” in 2012.

## 6 Practical Aspects of Fetal Growth Assessment in Japan

**FIGURE 4:**
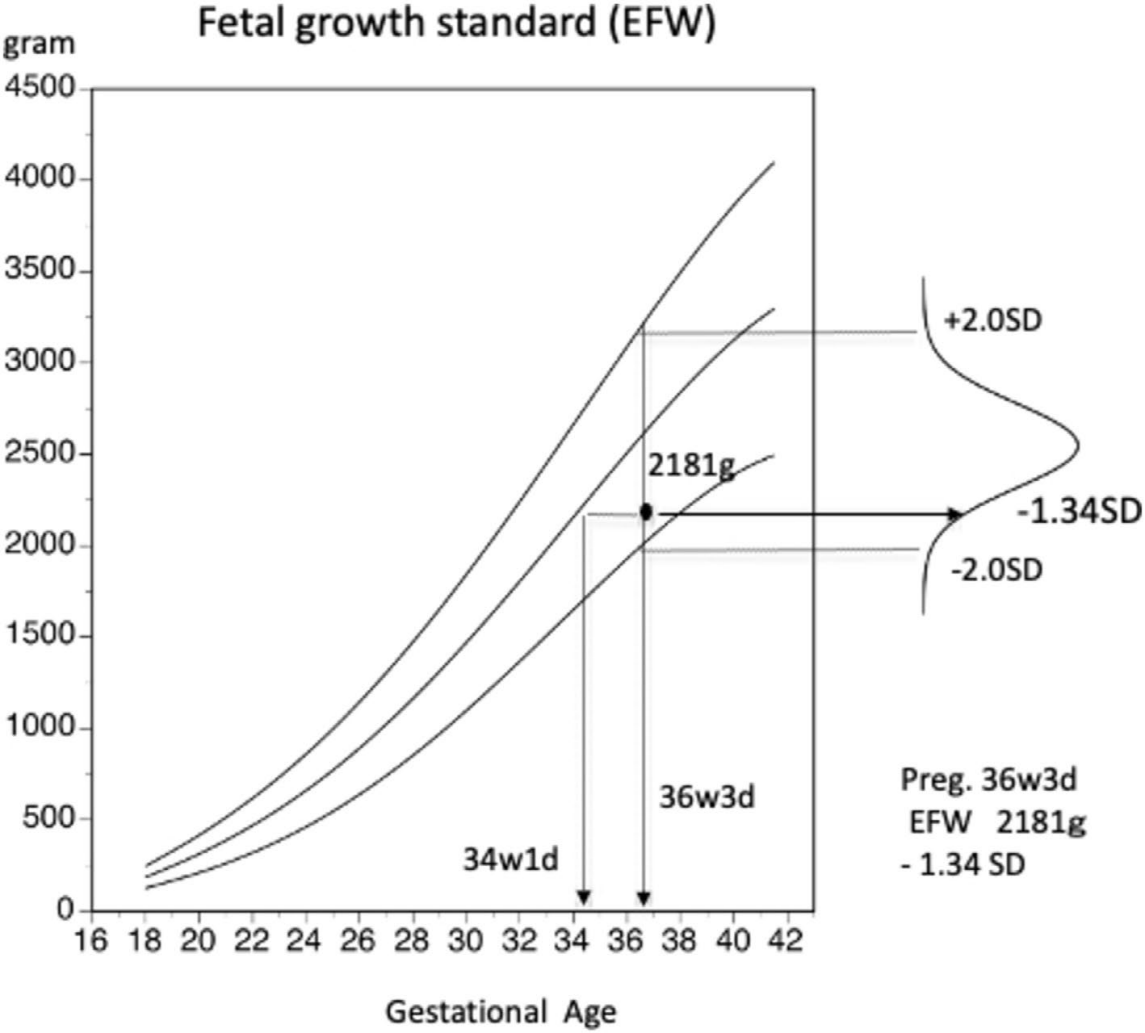
Quantitative description of fetal growth. Fetal growth is assessed relative to gestational age and should be expressed as gestational age with the corresponding EFW and standard deviation (e.g., 36 weeks 3 days, EFW 2181 g, −1.34 SD). Although an EFW of 2181 g corresponds to a mean gestational age of approximately 34 weeks 1 day, this “fetal age” has no predictive value for future growth or outcome.

Fetal growth is a function of gestational age; therefore, it should be expressed relative to gestational age using the EFW and its standard deviation (e.g., 36 weeks 3 days, EFW 2181 g, −1.34 SD). Since the reference standard values for fetal biometric measurements are derived from cohorts confirmed to follow a normal probability distribution, evaluations are expressed as deviations from the mean, rather than as percentile rankings Although an EFW of 2181 g corresponds to a mean gestational age of approximately 34 weeks 1 day, this so‐called “ultrasound fetal age” has no predictive value for future growth or perinatal outcome and should not be used clinically. Other fetal biometric parameters (such as BPD, AC, FL, and so on) should be evaluated in the same manner—that is, interpreted relative to gestational age using standard deviation, rather than being converted into an estimated gestational age (Figure 4). In Japan, this SD‐based method is commonly used in clinical practice. The EFW standard curve, including the ±2.0 SD range, is provided in the Maternal and Child Health Handbook. Because this range corresponds to the normal probability distribution, fetuses with EFW values within ±2.0 SD are regarded as within normal limits for gestational age and are expected to result in AFD neonates.

Numerous studies have investigated growth abnormalities, particularly fetal growth FGR, in relation to perinatal prognosis. Although SGA at birth may suggest the presence of FGR, FGR and SGA are not inherently equivalent concepts.

**FIGURE 5:**
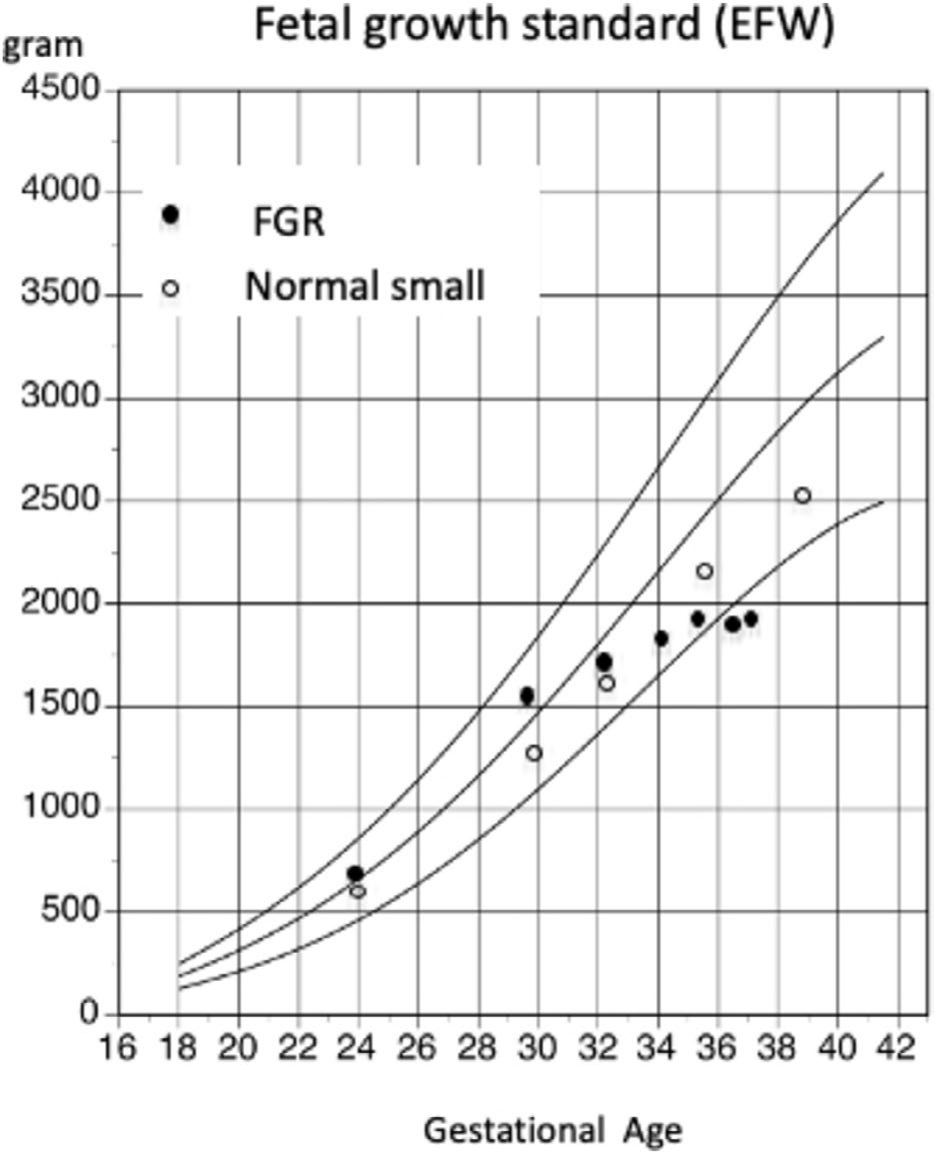
Chronological evaluation of fetal growth. Serial measurements of EFW reflect in utero growth velocity. A pattern demonstrating growth arrest (indicated by closed circles) is a key finding in the clinical diagnosis of FGR. Visualizing the data in a chart clarifies growth trends at a glance, while analyzing deviations over time offers a more rigorous, quantitative understanding of how those trends evolve.

In obstetric practice, ultrasound‐derived parameters, including EFW, are indispensable for the prospective assessment and management of fetal growth, allowing early identification of deviations from expected normal growth patterns. Consequently, FGR does not invariably lead to a SGA neonate at birth. While various criteria have been proposed for the diagnosis of FGR, in Japan, following the establishment of standardized normal reference values, a threshold of −1.5 SD has been widely adopted as a diagnostic criterion. Furthermore, in Japan, it is standard practice to perform multiple prenatal checkups (often 10 or more) and ultrasound examinations during pregnancy; therefore, it is essential to assess the temporal progression of fetal growth through serial measurements. The diagnosis of fetal FGR should not be based solely on measurements obtained at a single gestational age, but should also incorporate evaluation of chronological changes [18], namely growth velocity (Figure 5). However, no consensus has been established regarding the criteria for the optimal timing of identifying fetuses at high risk of group of FGR [19, 20].

The present discussion focuses on fetal growth; however, in clinical practice, functional maturity is also of critical importance. Accordingly, attempts have been made to perform comprehensive assessments incorporating additional parameters, such as fetal blood flow measurements, fetal movement, and amniotic fluid volume. Indeed, in Western countries, Doppler blood flow assessment is included in the diagnostic criteria for fetal growth FGR even when evaluation is based on a single gestational age [21].

## 7 Discussion

FGR is generally defined as a condition in which the fetus's intrinsic growth potential is compromised by internal or external factors. Its etiology is multifactorial, including congenital anomalies, genetic factors, infections, and alterations in the intrauterine environment associated with maternal complications, preeclampsia, or abnormalities of the placenta or umbilical cord, to which the fetus responds adaptively, resulting in growth restriction. Since the early reports by Gruenwald et al. [22, 23, 24], extensive studies on the diagnosis and management of FGR have been conducted, motivated by clinical concerns such as perinatal mortality, prevalence, and long‐term prognosis. Determining whether intrauterine fetal growth is normal represents the first step in assessing fetal health, and numerous fetal measurement methods, growth curves, and EFW formulas have been developed primarily for the detection of growth abnormalities.

SGA historically used to classify neonatal growth status has in recent years also been adopted in Western countries as a diagnostic term in the prenatal (in utero) setting. Based on the above considerations and from a historical perspective, my views on the concepts of SGA and FGR are summarized in Table 5. As the term “age” implies, SGA originally refers to the relative position of a measurement within a population‐based cohort at a single point in time, typically at birth. It is therefore a cross‐sectional and static definition. In contrast, FGR, as implied by the term “growth,” is a concept that should inherently be assessed over time. It reflects an evaluation of growth potential and the identification of reduced growth velocity. Accordingly, FGR should not be diagnosed based on a single data point but rather assessed through longitudinal (chronological) measurements, representing a dynamic definition.

**TABLE 5:** Opinions regarding the terminology and basic concepts of fetal SGA and FGR.

SGA
Static definition
Indicates the relative growth position of a measurement within the population base at a specific gestational age
FGR
Dynamic definition
Indicates a state of growth deceleration or arrest, representing growth potential;

SGA, historically used to define neonatal growth status, has more recently been adopted as an in utero diagnostic concept in the West. As the term implies, it represents a cross‐sectional diagnosis based on the distribution of measurements at a given point in time. While neonatal SGA is typically defined using a population‐based cohort (Table 1a), the cohort used for prenatal SGA assessment in other countries does not follow a normal probability cohort, but rather resembles those shown in Table 1c. Accordingly, cutoff EFW and abdominal circumference (AC) are determined using percentile‐based criteria.

In Western countries, differences in healthcare systems and screening protocols may preclude the accumulation of strictly controlled reference data, as achieved in our clinical context, necessitating reliance on more general descriptive reference values [9, 25], such as those presented in Table 1c. Several issues remain debated in the construction of growth charts. Although data variability is widely acknowledged, the accuracy of gestational age determination is also a fundamental concern; however, this aspect has been comparatively underemphasized in the literature.

In Japan, an established prenatal care system—including the Maternal and Child Health Handbook and coordinated regional healthcare networks—facilitates continuous management from the fetal to the neonatal period, with frequent ultrasound assessments conducted throughout pregnancy. Furthermore, a society‐based database enabling longitudinal tracking of developmental trajectories has been developed. As a result, data could be collected under the stringent conditions described in Table 2, allowing the establishment of a normal control survey cohort as the reference standard through rigorous statistical analysis. The scientific significance of the Shinozuka/JSUM reference values is therefore evident. By defining clear cutoff values for normal and abnormal, and by evaluating the temporal progression of deviations from the mean, changes in growth velocity can be inferred. The range of these normal values represents the underlying probability density and can be interpreted as corresponding to the likelihood of AFD birth based on the measured values.

For more than two decades, fetal growth has been evaluated in our clinical practice using accurately established gestational age and precise estimated EFW calculations based on reference values derived from rigorously defined normal control cohorts. While international standards, including those established in the United States, provide important frameworks, they should not be adopted uncritically. Rather, it is essential to develop and disseminate guidelines that align with our high standards of clinical practice [26, 27].

For raw fetal biometry data, please refer to the following site: https://shinozuka.com/US/usdata.html.

## Author Contributions

**Norio Shinozuka:** investigation.

## Funding

The author has nothing to report.

## Disclosure

The author has any financial interest with companies.

## Conflicts of Interest

The author declares no conflicts of interest.

## Data Availability

The data that support the findings of this study are available on request from the corresponding author. The data are not publicly available due to privacy or ethical restrictions.
